# Addressing the mental health needs of adolescents in South African communities: a protocol for a feasibility randomized controlled trial

**DOI:** 10.1186/s40814-021-00803-5

**Published:** 2021-03-16

**Authors:** K. Sorsdahl, C. van der Westhuizen, M. Neuman, H. A. Weiss, B. Myers

**Affiliations:** 1 Alan J. Flisher Centre for Public Mental Health, Department of Psychiatry & Mental Health, University of Cape Town, Cape Town, South Africa; 2 MRC Tropical Epidemiology Group, Department of Infectious Disease Epidemiology, London School of Hygiene and Tropical Medicine, London, England; 3 Alcohol, Tobacco and Other Drug Research Unit, South African Medical Research Council, Cape Town, South Africa; 4 Department of Psychiatry & Mental Health, University of Cape Town, Cape Town, South Africa

## Abstract

**Background:**

Like many low- and middle-income countries, almost half of the proportion of the South African population is under the age of 25. Given the peak age of onset for most mental health problems is in adolescence, it is vital that adolescents have access to mental health counselling. There are several initiatives to increase access to mental health counselling in South Africa, primarily through the integration of counselling for common mental disorders (CMD) into primary health care services, but adolescents (15–18 years of age) generally do not utilize these services. To address this gap, we will undertake a study to explore the feasibility of conducting a trial of the effectiveness of a community-based mental health counselling intervention for adolescents at-risk for a CMD.

**Methods:**

The study is a feasibility trial of the ASPIRE intervention, a four-session blended multi-component counselling intervention adapted for South African adolescents at risk for depression and alcohol use disorders. We will enrol 100 adolescents from community settings and randomly assign them to the ASPIRE intervention or a comparison condition. Feasibility measures, such as rates of recruitment, consent to participate in the trial and retention, will be calculated. Qualitative interviews with participants and counsellors will explore the acceptability of the intervention. The primary outcomes for a subsequent trial would be reductions in symptoms of depression and days of heavy drinking which will be measured at baseline, 6 weeks, and 3 months post-randomization.

**Discussion:**

This feasibility trial using a mixed-methods design will allow us to determine whether we can move forward to a larger effectiveness trial of the ASPIRE intervention.

**Trial registration:**

The trial is registered with the Pan African Clinical Trials Registry (PACTR20200352214510). Registered 28 February 2020—retrospectively registered, https://pactr.samrc.ac.za/TrialDisplay.aspx?TrialID=9795

## Background

Low- and middle-income countries (LMICs) are home to more than a billion adolescents, with a high proportion of adolescents and young people living in Africa [1]. Mental and substance use disorders are among the leading causes of years lived with disability (YLDs) among children and youth up to the age of 24 years globally, accounting for 25% of YLDs in this age group [2]. Early intervention for these disorders is critical given an individual’s health and behaviors in childhood and adolescence lay the foundation for health in later years, and impact on the health of their offspring [3]. Moreover, the well-being of adolescents is integrally linked to socio-economic development in LMICs [4], including South Africa.

Like many LMICs, there is a significant mental health treatment gap in South Africa. Approximately 30% and 17% of adults in South Africa meet diagnostic criteria for a lifetime and past year mental disorder respectively, yet less than a quarter ever receive treatment [5]. There have been several initiatives to increase access to mental health counselling in South Africa, primarily through the integration of community health worker-delivered counselling for common mental disorders (such as depression and alcohol use disorders) into primary health care (PHC) services [6–8]. While these efforts are starting to reduce the treatment gap for adults who access PHC services, adolescents rarely use health services offered on the primary care platform. Where youth-friendly clinics are available, these do not offer mental health counselling. Moreover, data suggests that at-risk adolescents globally and in South Africa under-utilize mental health services [9, 10] with only 30% of this at-risk population seeking help for mental health problems [11].

Failure to reach adolescents is a major service and treatment gap given this is the peak age of onset for most mental health problems. If left untreated, these problems predict adverse life trajectories [12]. Adolescents who do not seek or access mental health services run the risk of developing more advanced psychopathology in adulthood [13, 14] and are at greater risk for physical health comorbidities and injury [15]. For example, heavy alcohol use during adolescence may contribute to unsafe sex practices that increase the likelihood of unplanned pregnancies and sexually transmitted infections, such as HIV [16]. Addressing mental health problems at an early stage may also alleviate the long-term costs to society and the public health system of untreated mental disorders [17].

While there is some promising evidence for effective interventions to promote mental health among adolescents globally [18–22], available evidence from South Africa for the effectiveness of these interventions is primarily focussed on adults. There is evidence from South Africa to suggest that brief, structured counselling interventions, when task-shared to trained community health workers (CHWs), may improve mental health outcomes among adults [8]. Motivational interviewing (MI), behavioral activation, and problem-solving therapy (PST), are recommended interventions from the World Health Organization’s programme to reduce the mental health treatment gap [23]. In South Africa, 3–4 sessions of counselling that included elements of these recommended interventions improved depression and alcohol outcomes among patients presenting to healthcare facilitates [8]. There is good evidence that combining MI and cognitive behavioral treatments (CBT) leads to improved outcomes for people with depression [24, 25], alcohol and other substance use disorders [8], as well as those with comorbid alcohol use disorders and depression [26]. Despite both MI and CBT approaches (such as PST and behavioral activation) being used to intervene with adolescents in high-income countries [21, 27, 28], this blended multi-component counselling intervention had not yet been tested among this population in South Africa.

This paper describes the study protocol for a feasibility trial of the ASPIRE intervention, a multi-component counselling intervention for South African adolescents at risk for a common mental disorder (specifically depression and alcohol use disorders). This feasibility trial aims to provide the early evidence needed to help service planners develop and implement adolescent-oriented counselling. It is hoped that the findings from this feasibility trial will lay the groundwork for a future effectiveness trial of the ASPIRE intervention.

### Feasibility trial objectives

In this feasibility trial, standard trial procedures will be implemented to obtain information on the acceptability and feasibility of the ASPIRE intervention for South African adolescents, including safety considerations. We will also assess the feasibility of our study methods and counselling, training and supervision model that has been adapted for this population [29]. Using a mixed-methods design with qualitative and quantitative approaches, this feasibility trial will allow us to identify and refine issues pertaining to recruitment, retention, intervention delivery, data collection, randomization, and blinding prior to a future effectiveness trial.

The primary objectives are to (i) test the feasibility and acceptability of the ASPIRE intervention as well as (ii) the feasibility of all study procedures and performance of outcome measures to inform a fully powered future trial. A second objective is to explore the initial effect of this intervention on days of heavy drinking and symptoms of depression.

## Methods

This protocol is reported in accordance with the guidelines presented in the Standard Protocol Items: Recommendations for Interventional Trials (SPIRIT) checklist [30] and the Template Intervention Description and Replication guidelines [31].

### Trial design

**Fig. 1:**
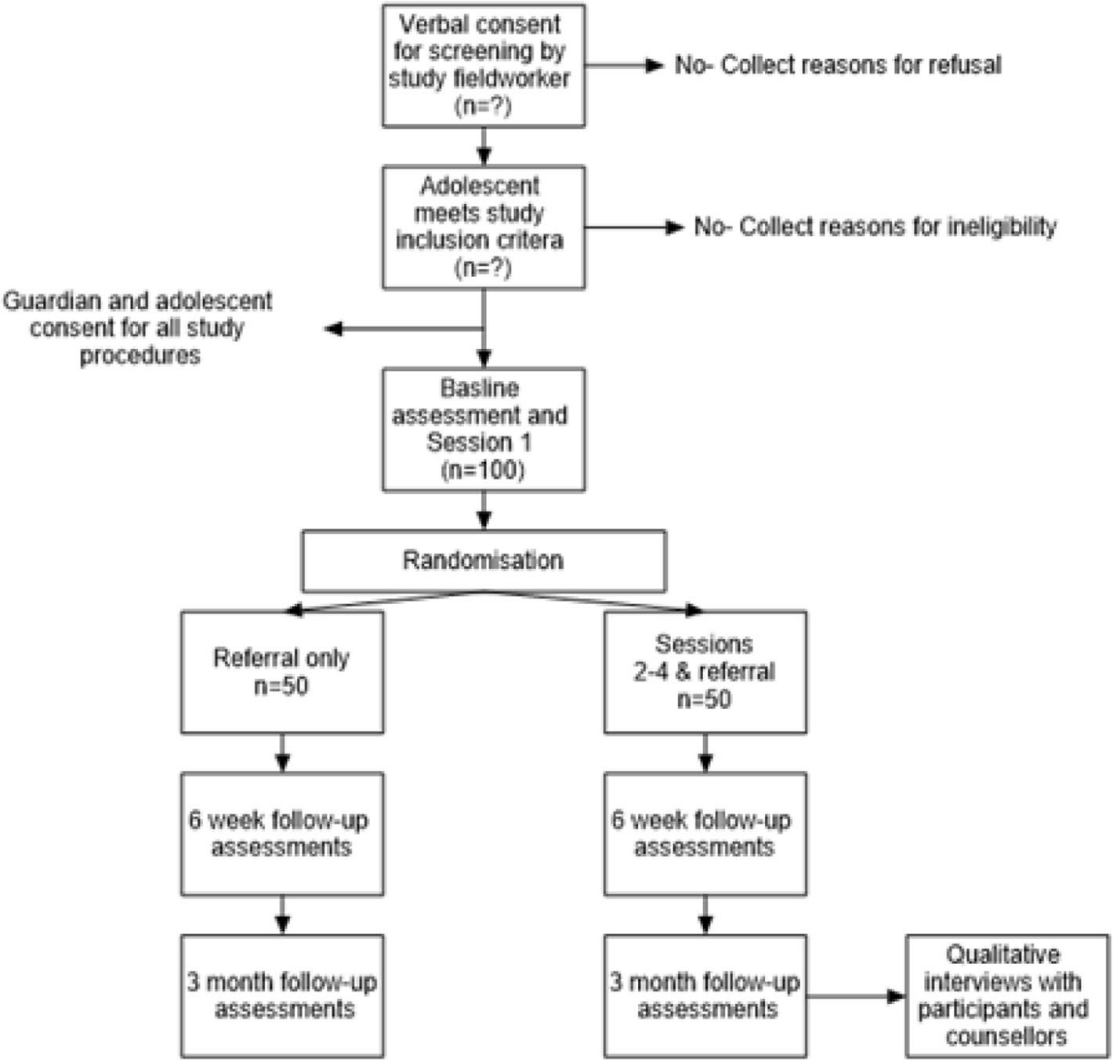
Consort diagram of the study design

An individually randomized two-arm feasibility trial, with embedded qualitative interviews will be conducted (see Consort diagram in Fig. 1). We will enrol 100 adolescents from community settings and randomly assign them to the ASPIRE intervention or a comparison condition. Participants will be tracked for 6-week and 3-month post-randomization follow-up assessments.

### Study setting

We will recruit participants from a mix of communities in and around Cape Town within the Western Cape (WC) province of SA. These communities are characterized by high levels of unemployment (> 70%) and CMDs among young people [32]. Study activities will take place at the South African Medical Research Council’s clinical research site in Delft, which is within easy access of several of these communities and at community-based organizations that provide services for at-risk adolescents and their families.

### Participants

To be eligible for inclusion in this study, participants must (1) be aged between 15 and 18 years old; (2) provide written informed assent/consent to participate in the study; (3) have written informed parental consent to participate if younger than 18 years of age; (4) screen at risk for depression with a score ≥ 10 on the Center for Epidemiology Studies Depression Scale short form (CES-D-10 [33];) and/or screen at moderate or severe risk for alcohol-related health problems, with a score ≥ 5 on the Alcohol, Smoking and Substance Use Involvement Test-Youth (ASSIST-Y [34]); and report at least 2 episodes of heavy drinking (≥ 5 standard drinks on a single occasion) in the last month. Participants will be excluded if they are currently receiving any form of treatment for a mental or substance use disorder.

### Participant recruitment and study procedures

We will use established community-based outreach techniques in organizations and places that adolescents frequent to identify potential participants. These methods were used successfully in previous studies to recruit larger samples of vulnerable populations, including youth [35]. While all contact with adolescents - including assessments and counselling sessions - has been planned as occurring face-to-face, the team may conduct telephonic recruitment, assessments or counselling sessions where necessary.  Fieldworkers will approach potential participants in various community settings. After explaining the purpose of the study, the fieldworker will request verbal consent to screen the adolescent for possible study inclusion. If the adolescent is not eligible, they will be thanked for their time and given health information materials. If they are eligible, but do not provide consent, reasons for lack of interest will be recorded. Where parental consent is not required, an appointment will be made for a study enrolment visit with eligible and interested adolescents. If parental consent is required, the fieldworker will contact the parent to obtain this prior to making an appointment for an enrolment visit. The fieldworker will follow a script for this discussion with the adolescent’s parent and will not disclose the mental health criteria on which their child was eligible. If the parent refuses to consent, the adolescent will not be enrolled.

At the enrolment visit that will take place at the South African Medical Research Council’s (SAMRC) clinical research site in Delft, the fieldworker will re-screen the participant to confirm study eligibility prior to obtaining written informed consent/assent to participate in the trial. Thereafter the fieldworker will administer the baseline assessment in either English, Afrikaans, or isiXhosa, the three official languages of the Western Cape province. The assessment includes socio-demographic information as well as information on mental health and behavioral problems, family relationships; and social support. All participants will then be matched with a trained ASPIRE counsellor to receive a counselling session. After counselling is completed, the trial manager will assign the participants to the intervention or comparison group. The counselling session will take place the same day as enrolment. A further three counselling appointments will be scheduled for adolescents allocated to the intervention group. These will be spaced at least a week apart from each other. Participants will have six weeks from enrolment to complete all four sessions, whereafter a participant will time out of the intervention. Irrespective of study arm, all participants will be asked to return for follow-up assessments at 6-week and 3-month post-enrolment. During these follow-up visits, a fieldworker will re-administer the baseline assessments. Participants will have 30 days from their scheduled appointment to complete these follow-up assessments before timing out of that appointment.

A random sample of approximately 30 adolescents who received the ASPIRE intervention will be recruited to participate in qualitative interviews to explore their perceptions of (1) intervention acceptability and suggestions for modification; (2) feasibility of study procedures; and (3) barriers and facilitators to participation and retention in counselling. These will take place after their final follow-up assessment. Counsellors delivering the intervention will also be interviewed to examine their experience of delivering the intervention and to identify any barriers and facilitators to intervention delivery. Transport or costs associated with transport required to attend study or counselling appointments will be provided or reimbursed. Participants will receive grocery vouchers for completing the baseline and follow-up assessments.

### Randomization and blinding

Participants will be randomly allocated in a 1:1 ratio to either the intervention or comparison arm. The randomization sequence will be prepared by the data manager (using a computer programme) and allocation will be done by a trial manager. The fieldworkers will phone the trial manager when an individual is ready to be allocated, and the trial manager will inform the fieldworker about the participant’s allocation based on the randomization sequence. The fieldworker will then notify the participant about his/her allocation and associated activities. Investigators will be blind to the sequence generation. The trial manager will have no direct contact with participants, and all allocations will be concealed until the individual is assigned to a study arm. To reduce potential performance bias, allocations will only occur after the first counselling session has been completed. Fieldworkers will conduct the baseline and follow-up assessments, and trained counsellors will deliver the intervention. Fieldworkers conducting the follow-up assessments will be blinded to the randomization arm. Counsellors delivering the intervention and fieldworkers (serving as outcome assessors) will function independently of each other. Counsellors will not conduct any assessments, ensuring that these assessments remain independent from the counselling sessions.

### Interventions

Irrespective of study arm, the interventions will be delivered in a face-to-face format by trained and experienced health counsellors. These counsellors are increasingly being used in health and community settings in South Africa to deliver structured psychosocial interventions and are part of the National Department of Health’s plans to scale up the provision of mental health counselling through task-sharing structured interventions. We have successfully used these counsellors to deliver other psychosocial interventions and have shown that patients prefer lay counsellors to whom they can relate rather than other health workers [36, 37]. See Table 1 for a detailed description of the ASPIRE intervention package.

**Table 1:** Components of the ASPIRE intervention package

Theoretical framework (intervention)	Lazarus and Folkman’s coping theory (Lazarus and Folkman, 1984) informs the problem-solving therapy (PST) approach: problem-solving for problems that can be solved and emotion-focused coping for problems that can’t be solved
**Delivering agents**	• Training in generic counselling skills and 2–5 years of counselling experience
**Structure of intervention package**	Four sessions of blended multi-component counselling intervention, to be delivered weekly. Participants have a 6-week window to complete all sessions before timing out of the intervention.
**Structure of sessions**	
***Session #1*** (participants in both arms receive this)	• Conduct screening/assessment of mental health • Provide feedback on results of screening/assessment • Increase knowledge of how depression and/or alcohol use impacts on adolescents • MI to build rapport and develop readiness to change (for alcohol) • Behavioral activation for strategies to address symptoms of depression
***Session #2*** (intervention arm only)	• Patient check-in using MI • Build the rationale for PST o Explain the structure and rationale for PST o Establish positive problem orientation • Teach the steps of PST • First problem-solving exercise with counsellor and homework
***Session #3*** (intervention arm only)	• Patient check-in using MI • Review practice exercises from session 2 and discuss challenges • *Coping with negative thoughts*: explain how to cope with problems that are not important • Second problem-solving exercise with counsellor and an exercise • *Emotional regulation* • Explain how to cope with “big” feelings such as anger • Practice emotional regulation techniques • Third problem-solving exercise with counsellor and homework
***Session #4*** (intervention arm only)	• Patient check-in using MI • Review practice exercises from session 3 and discuss challenges • *Advance process of acceptance*: teach how to deal with problems that are important and cannot be solved • Fourth problem-solving exercise with counsellor and recap • *Bringing it all together*
**ASPIRE training**	
**Structure and format of training**	• Forty hours of formal training (the equivalent of five working days) • Mixture of didactic teaching and experiential group activities including skills rehearsal exercises and role plays • Counselling proficiency assessed during role plays using a competency checklist
**Training content**	• Understanding common mental disorders (CMDs) • Principles of counselling (including confidentiality) • Screening participants for hazardous/harmful alcohol use and depression • Delivery of the blended multi-component counselling intervention • Ethics: recognizing and managing distressed participants and referral for additional care
**Characteristics of supervisor**	• Psychological counsellor, registered with the HPCSA • Five years previous counselling experience in cognitive-behavioral therapy-based brief interventions • Three years previous experience in delivering MI-PST and training healthcare workers • Conduct guided by the professional standards and ethics–Professional Board for Psychology and the HPCSA
**Structure of supervision and debriefing**	• In-person or telephonic individual supervision and debriefing conducted once a week • Telephonic supervision and debriefing used during community unrest, gang violence • Brief communication via text or WhatsApp messaging to address challenges in real-time in between weekly scheduled supervision and debriefing sessions • Supervision and debriefing of up to an hour per session and structured as follows: o Debriefing: brief check-in, followed by reflection on recent experiences at work and/or home, how these experiences were dealt with emotionally and practically (coping) and identifying opportunities for growth o Clinical supervision: counsellors present new cases and/or discuss patient progress including feedback, suggestions or recommendations by supervisor where needed in addition to reviewing counselling session notes o Addressing challenges: discuss logistical and counselling delivery challenges, and brainstorm solutions o Counselling fidelity feedback: provide counsellors with structured feedback on their counselling proficiency using a counselling fidelity checklist (audio tapes of counselling sessions assessed prior to supervision by supervisor). Brief skills rehearsal exercises or role playing to improve and solidify counselling aspects with average to low scores on the fidelity checklist.
**Supervisor training and support**	• Trained to use a structured approach to supervision • Weekly in-person or telephonic supervision provided by a psychologist to assess adherence to the supervision approach and discuss ways of overcoming any logistical and systemic challenges to provision of supervision and debriefing • Weekly in-person or telephonic debriefing provided by a psychologist, including reflecting on recent experiences at work and/or home, how these experiences were dealt with emotionally and practically (coping) and identifying opportunities for growth

#### Comparison arm: session 1 and referral to care

Participants assigned to this arm will have received a single counselling session (~ 1 h in duration) prior to allocation. This is session one of the ASPIRE intervention, and the content and delivery of this session will be identical to that received by participants assigned to the intervention condition. The session includes screening for heavy alcohol use and depression, feedback about the screening results and levels of risk, psycho-education about how alcohol or feelings of depression can affect young people, and (depending on their primary condition), motivational interviewing to enhance motivation to reduce alcohol use or behavioral activation to address symptoms of depression. The session will conclude with the participant setting personal goals and developing a plan for change, guided by the counsellor. Participants will also be given referrals to usual care providers for further follow-up if required.

#### Intervention arm (ASPIRE intervention)

**Fig. 2:**
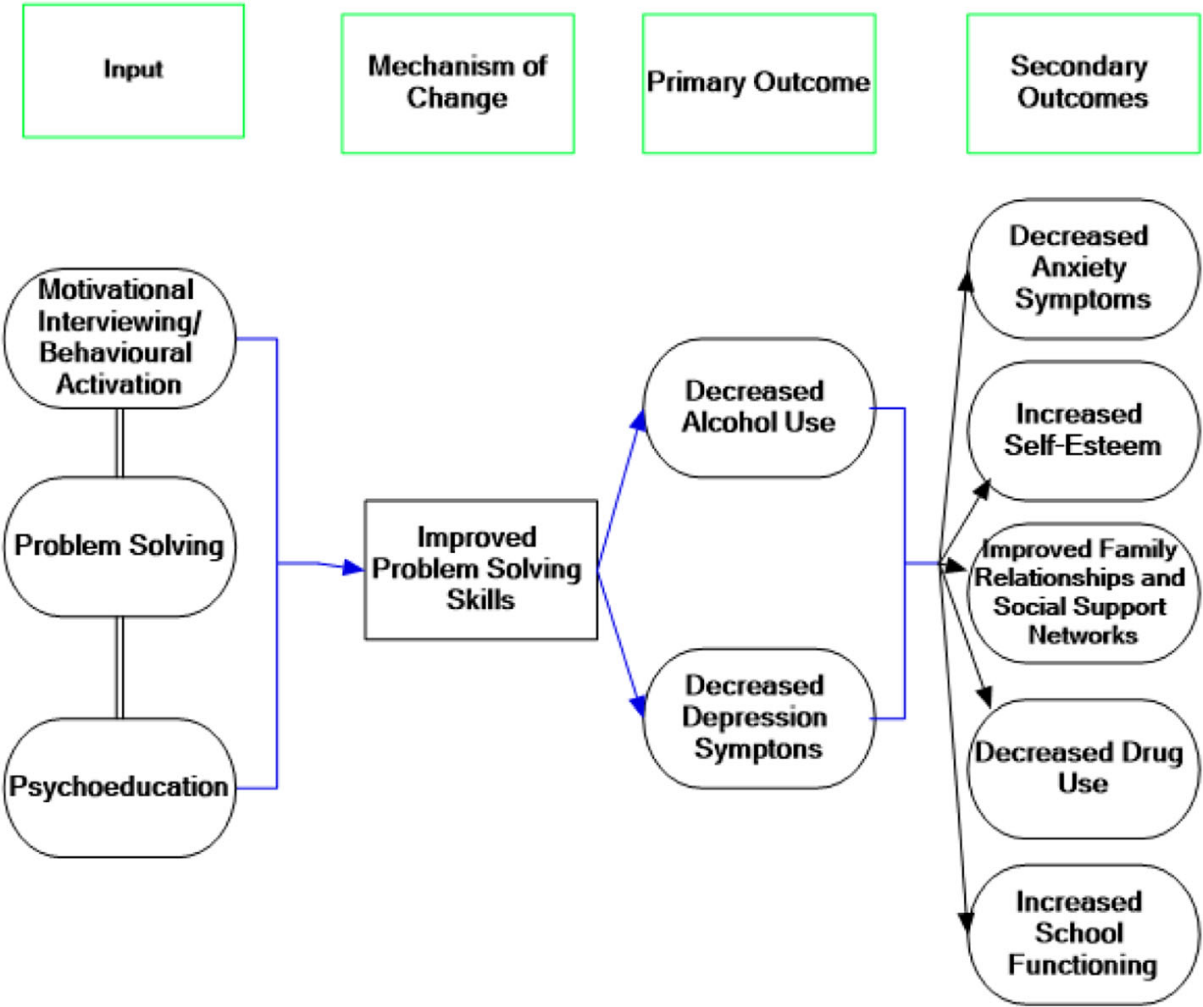
Conceptual model of the ASPIRE intervention

Participants assigned to this condition will receive session one as described above, plus an additional three sessions of  a blended multi-component counselling intervention that has shown efficacy in other studies [8]. The goals of this intervention are to motivate participants to make changes to their risk behaviors and strengthen their problem-solving skills to help them cope better with stress and life problems that are risk factors for common mental disorders. The intervention is largely based on Lazarus and Folkman’s [38] coping theory, teaches problem-focused coping skills for mutable problems and emotion-focused coping strategies (acceptance and seeking support) for immutable problems. In this approach, heavy alcohol use is viewed as a form of problem avoidance (maladaptive emotion-focused coping) and depression, a response to maladaptive emotion-focused coping [38]. See Fig. 2 for a conceptual model of the ASPIRE intervention. Each session functions iteratively to build readiness to change and adaptive problem-solving and coping skills. All sessions have a motivational component, a psycho-education component (in which participants are taught problem-solving skills and how to apply them) and include an opportunity to apply newly learned skills through exercises and homework. More specifically, participants are taught steps for addressing problems that are important and can be solved; strategies for dealing with negative and intrusive worries that are unimportant; and steps for coping with problems that are important but cannot be solved [8, 39]. Participants will also be taught techniques for managing uncomfortable emotions. A patient handbook, summarizing the content of the counselling sessions and containing worksheets that participants use to practice the problem-solving method, is used to guide counselling. The handbook is available in English, Afrikaans, or isiXhosa (the three official languages of the Western Cape). From enrolment, participants in the intervention arm will have 6 weeks to receive all four sessions of the intervention. Each counselling session should be spaced at least 5 days apart, with the first session occurring immediately after the baseline assessment. The duration of each counselling session is approximately 45–60 min.

### Counsellor training and supervision

ASPIRE counsellors selected to be trained will already have basic counselling training. For this study, they will receive an additional 40 h of training in understanding common mental disorders, screening for hazardous/harmful alcohol use and depression, principles of basic counselling (including confidentiality), motivational interviewing and problem-solving therapy (with proficiency testing through role-playing and case examples), providing referrals, and responding to distressed participants. Prior to the start of training, counsellors will complete an assessment of their knowledge, attitudes, beliefs, and practices around counselling for common mental disorders, and these will be assessed after completion of training to evaluate the impact of training.

Following training and continuing throughout the implementation of the interventions, counsellors will participate in regular face-to-face and virtual supervision and debriefing that will be conducted by a registered psychological counsellor (see Table 1 for further details). The content of supervision will include review of the logistics of implementing the intervention (to troubleshoot any barriers), a review of participants’ progress and debriefing about challenges, feedback from fidelity checks about the quality of intervention delivery, and how it can be improved. Counsellors will take notes after each counselling session, which will be reviewed during supervision by the counsellor supervisor. Supervision will also be an opportunity for on-going booster training, as and when required. If quality assurance activities indicate that some counsellors require more training in the intervention, this will be provided. The number of hours spent on training, supervision, and debriefing per individual counsellor will be recorded in a training and supervision log. The counsellor supervisor in turn will be supervised by a psychologist. Supervision sessions will be recorded for fidelity and reviewed, with feedback provided to improve the quality of supervision. We have used this model of supervision successfully in our previous work [29].

### Counsellor competency and monitoring

To assess and monitor counsellor competency in both arms, we will audio-tape counselling sessions with consent from participants. For the comparison arm, counsellors’ competency in delivering session 1 will be assessed. For participants in the intervention arm, one session will be randomly selected from the total number of completed sessions to be assessed for counsellor competency. Both treatment-specific competencies and core therapeutic competencies will be assessed by the counselling supervisor.

For treatment-specific competencies (often referred to as fidelity), an intervention delivery checklist will be used which includes items pertaining to (1) use of a motivational interviewing counselling style; (2) dosage (duration of counselling session and amount of time spent on non-intervention related topics); and (3) whether the objectives of the intervention session were addressed adequately. For this project, we will rate these competencies using Likert scales, with 1 being “not at all present” and 5 being “present most of the time. For core competencies, a South African version of the ENhancing Assessment of Common Therapeutic Factors [40] will be used to measure core therapeutic competencies and skills that are thought to be required for a counsellor to adequately deliver any evidence-based intervention.

### Feasibility outcomes

**Fig. 3:**
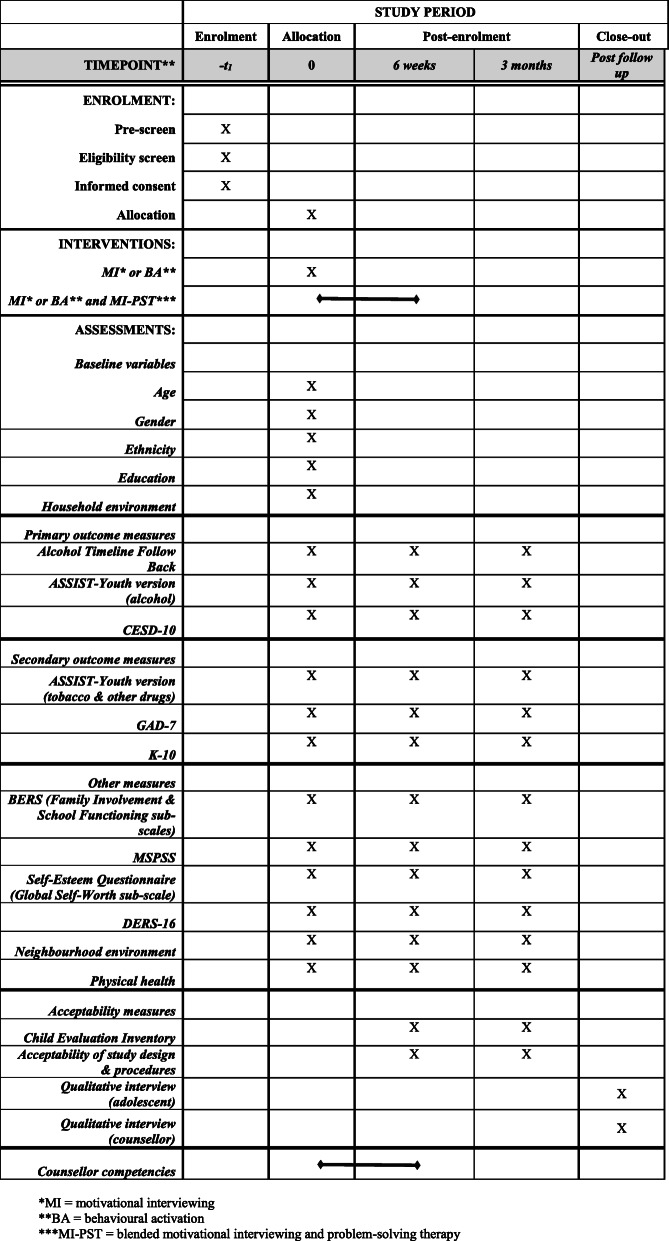
Standard Protocol Items: Recommendations for Clinical Trials (SPIRIT) Figure–schedule of data collection

The schedule of data collection is shown in (Fig. 3) the SPIRIT Figure. The primary objective of this study is to evaluate the feasibility and acceptability of the ASPIRE intervention, and the trial procedures required for the implementation of a future effectiveness trial. In line with recommendations for the reporting of pilot studies [41], we have identified several quantitative and qualitative indicators of feasibility and acceptability that will be used to guide decisions about what procedures to carry through to the full trial and when modifications to procedures or intervention content and delivery should be made. These include (1) feasibility of recruitment; (2) appropriateness of data collection processes and outcome measures; (3) retention in the ASPIRE intervention; (4) counsellor competency; (5) feasibility of randomization and blinding; (6) presence of adverse advents; and (7) acceptability of the ASPIRE intervention and study procedures. Table 2 describes these indicators, how they will be evaluated and pre-determined progression criteria (where applicable). Progression criteria have been set to facilitate the interpretation of results and to inform whether to proceed to a definitive trial after the feasibility study.

**Table 2:** Overview of feasibility and acceptability outcomes and progression criteria

Outcome	Evaluation	Progression criteria to controlled trial^**a**^
**Feasibility of recruitment**	• Numbers screened, number of eligible participants, number invited to participate, consent rates (for parents and adolescents), refusal rates and reasons for refusal	• Enrolment of at least 5 participants per week
**Appropriateness of data collection processes and outcome measures**	• Number of missing items and follow-up rates	• Less than 15% missing items for each measure
**Retention in the ASPIRE intervention**	• Number of participants who completed at least three sessions	• At least 70% of participants complete the counselling programme
**Counsellor competencies in delivering the ASPIRE intervention**	• Scores on a counsellor competency checklist	• 75% competency
**Feasibility of randomization**	• Number of consent/assent • Number of refusals to be randomized	• At least 80% participation
**Feasibility of blinding**	• Field staff perceived contamination	• Very minimal perceived contamination (less than 5%)
**Presence of adverse events**	• Number of study-related adverse events reported	• Less than 10% of participants reporting severe adverse events related to the study
**Acceptability of the ASPIRE intervention**	• Qualitative interviews with participants and counsellors following the feasibility trial	• Not applicable
	• Scores on the Child Evaluation Inventory (CEI) used to assess participants’ satisfaction with the intervention and how it was delivered	• More than 80% of participants scored a total of 60 or more on the CEI
**Acceptability of the study design and procedures**	• Participants will be asked to rate the acceptability of the study procedures, including (i) screening, (ii) consent, (iii) assessment (length, type of questions, mode of administration), (iv) randomization, (v) referrals (and uptake of referrals), and (vi) tracking for follow-up assessments. They will rate these items on a 5-point scale (from 1 = not at all acceptable to 5 = very acceptable).	• More than 80% of participants score 3 or more for each rating

^a^If one or more criteria are not met revisions should be considered before proceeding to a definitive trial

### Measures to assess participant-level clinical outcomes

Although clinical outcomes in this feasibility trial are secondary to the feasibility outcomes, we plan to collect data on our planned outcomes for a future trial to establish the feasibility of using the proposed measures. Measures will be considered feasible if fewer than 15% of items are incomplete (see Table 2).

### Proposed primary trial outcome measures

All primary outcomes are at the level of the individual patient and will be collected at baseline and at the two follow-up assessments (see Fig. 3).

#### Percent of heavy drinking days in the past month

We will use the Alcohol Timeline Follow Back (TLFB) technique to collect self-reported frequency, quantity, volume, patterns, and types of alcohol consumption. This will be used to calculate the percent of heavy drinking days (> 60 g absolute alcohol) in the past month [42].

#### Alcohol severity

Alcohol use will be measured using Alcohol, Smoking and Substance Use Involvement Test - youth (ASSIST-Y) scores [34]. The ASSIST-Y for adolescents aged 15–17 will be used. This measure categorizes people into low, moderate, or high risk for substance-related problems. Those with alcohol scores < 5 are considered low risk, ≥ 5 and ≤ 17 are considered at moderate risk, and ≥ 18 are considered high risk. This outcome is restricted to adolescents who report alcohol problems at baseline.

#### Symptoms of depression

We will use the Center for Epidemiologic Studies Depression Scale (CES-D-10), a 10-item scale that measures depressive feelings and behaviors during the past week to assess change in symptom severity on this outcome. This outcome is restricted to those with CES-D > = 10 at baseline. The CES-D 10 has been validated for use in South Africa and with adolescents [43, 44] and has shown good psychometric properties. A cut-off score of 10 indicates risk of depression, with scores ranging from 0 to 30 [45].

### Secondary outcome measures

#### Coping and problem-solving

Social problem-solving will be assessed using the Social Problem-Solving Inventory - Adolescent version, short form (SPSI-A, [46]. The SPSI-A SF consists of 30 items organized into three scales: Automatic Process, Problem Orientation, and Problem-Solving Skills.

#### Tobacco and other drug use

We will use the ASSIST-Y [34] to assess use of tobacco and other drugs. The ASSIST-Y collects information on frequency of tobacco and other drug as well as severity of risk for tobacco and other drug-related problems.

#### Anxiety

We will use the generalized disorder scale (GAD-7) to assess the severity of self-reported symptoms of generalized anxiety. The scale has been shown to have good psychometric properties [47, 48].

#### Family relationships

We will use the 10-item Family Involvement subscale of the Behavioral and Emotional Rating scale to assess the adolescent’s relationship with their family [49, 50].

#### Self-esteem

This will be measured using the 8-item Global Self Worth subscale of the Self Esteem Questionnaire [51]. This sub-scale has demonstrated high validity and adequate psychometric properties in South African adolescents [52].

#### Social support

The Multi-Dimensional Scale of Perceived Social Support (MSPSS) [53] will be used to assess social support from family, friends and significant others. This instrument has been shown to have good concurrent, construct and discriminant validity, and high internal and test re-test reliability [53] and good reliability and validity with adolescents [54].

#### School involvement

We will use the School Functioning subscale (9 items) of the Behavioral and Emotional Rating Scale (BERS) to assess the adolescents’ performance and competence in school. This scale assesses aspects such as attention in class and completion of school tasks [50].

### Sample size considerations

As we are not testing effect sizes in this feasibility trial, a power calculation is not required. Recommendations for sample size requirements to estimate key design parameters from external feasibility and pilot randomized controlled trials suggest that at least 70 measured subjects (35 per group) are required when estimating the SD for a continuous outcome [55]. Therefore, we will recruit 100 participants (50 per group) to account for attrition.

### Data analysis

The quantitative outcomes of interest (see Table 2) will be summarized descriptively using appropriate summary statistics (mean and standard deviation for continuous outcomes and numbers and proportions for categorical outcomes) and presented graphically over time for both study arms.

We will explore clinical outcomes. In these exploratory analyses, we will calculate means and confidence intervals of clinical outcomes (both within- and between-study groups) and determine which outcomes are most sensitive to change. We will evaluate the parameters required to inform the sample size calculation for a future main trial. We will also assess change in clinical outcomes using an intention-to-treat analysis. Outcomes will be compared between intervention and control groups using linear regression models, adjusting for the baseline score of the given outcome. We will create separate models for alcohol and depression and only include participants who screen at risk at baseline. The two primary outcomes will be ascertained at three time-points for each participant. This repeated measure feature will be used to estimate the intervention effects and 95% confidence intervals for the two primary outcomes at 6 weeks and 3 months for the contrast between the intervention and comparison condition.

Qualitative data will be coded with NVivo and analyzed using the framework approach [56]. Transcripts of interviews will be read for emergent themes and then coded. Coding and analysis will continue iteratively. Two project staff will code the transcripts; they will meet after the first five transcripts, and thereafter after every 10 transcripts, to compare notes. Cohen’s Kappa will be used to measure inter-coder agreement. Following the Consolidated Criteria for Reporting Qualitative Studies (COREQ), we will document the process according to the 32-item checklist [57].

### Ethical considerations

The South African Medical Research Council (SAMRCEC 012-8-2018), the University of Cape Town (276/2018) and the London School of Hygiene and Tropical Medicine (17873) provided ethical approval for this study. The trial is registered with the Pan African Clinical Trials Registry (PACTR20200352214510).

#### Informed consent

Prior to eligibility screening, the fieldworker will briefly describe the study before requesting verbal consent to screen the person for possible study inclusion. We will not use the information collected on the screener for any purposes other than to describe reasons for study ineligibility. Parental consent and adolescent assent or consent will be obtained from all potential individual participants prior to enrolment. Prospective participants will be informed of all foreseeable risks of study involvement, that participation is voluntary and that they may withdraw their consent at any times. The consent and assent forms are available in English, Afrikaans, and isiXhosa, the main languages spoken in the region.

#### Confidentiality

The confidentiality of the participant will be respected and maintained at screening, recruitment, data entry, storage, analysis, and dissemination of findings stages. To ensure confidentiality, a unique participant identification number will link the various study forms. The study database will be password-protected following standard password safety procedures. The data manager will review these data daily for quality assurance and quality control purposes. All data will be stored in double-locked filing cabinets in designated locked offices. Forms with personal identifying information will be stored separately from case report forms. All participant case report forms will be kept in locked study-dedicated file cabinets at the SAMRC. Confidentiality will be broken in the case of threat of imminent harm to self or others and/or the abuse or neglect of a child. The informed consent forms state these exceptions to the promise of confidentiality. All fieldworkers and counsellors will be trained in how to manage issues of harm to self or others that a participant might raise during data collection or counselling. In these instances, participants will be informed about the need to breach confidentiality in such situations.

#### Anticipated risks

The main risk associated with this study is the potential mental discomfort that may arise from material covered in the assessments or intervention sessions. This will be minimized by using experienced and well-trained fieldworkers and counsellors and the option of referral to mental health and substance use services in both arms. Staff will be trained on how to identify distressed participants who may be at risk of harm and will be supported in managing these participants telephonically during the patient contact time, and during regular supervision and debriefing sessions. For participants who are distressed and at risk of harm, we will actively refer and link them to appropriate resources to help them cope and deal with their distress. A further risk shared with other psychotherapy interventions is initial worsening of symptoms and risk of suicide arising from issues uncovered during counselling. To minimize this potential harm, we will train staff to screen all participants who report or display signs of distress for risk of suicide (using the Columbia Suicide Severity rating [58] scale and to provide referrals to specialized mental health services.

All adverse events (AE) and serious adverse events (SAEs) that are reported by the participant or observed by the field workers or counsellors will be recorded. All staff will be trained to follow the Distressed Participant Protocol that provides the process of reporting and supporting/referral in case of any adverse events. All AEs and SAEs will be reported to a local independent Trial Steering Committee (TSC).

### Trial management

A trial management team (TMT) will be formed comprising the PIs, other investigators, the counselling supervisor and the trial manager. The TMT will be responsible for the day to day running of the trial and will meet approximately once a week during the trial. The TMT will provide feedback on trial progress to the Trial Steering Committee (TSC) that will be convened to provide overall supervision of the trial, ensure its conduct is in accordance with the principles of relevant regulations, and monitor data and trial safety.

## Discussion

This study is likely to contribute to the small body of global evidence on promising trans-diagnostic counselling interventions for at-risk adolescents. First, although a number of studies in LMICs have explored interventions for the prevention and treatment of mental health problems in adolescents, most of these interventions were delivered in school settings and focus on a single disorder, namely behavioral problems [59] or post-traumatic stress disorder (PTSD) [60, 61], with only a few interventions targeting depression [62]. Further, the evidence for the effectiveness of brief interventions for problematic alcohol use is limited [63] with no available data from LMICs. Alcohol use and depression are major issues among adolescents in the Western Cape. A provincial school survey of 10,301 learners found that 25% of the adolescents who reported lifetime alcohol use, were heavy episodic drinkers and 56% of learners were at moderate to high risk of mental health problems [32, 64]. Given this blended multi-component counselling intervention has previously demonstrated acceptability, feasibility, and efficacy in adults with CMDs [8, 65], we believe it has the potential to be similarly beneficial for adolescents following necessary age-appropriate modifications to content and delivery approaches, which were completed during the formative work for this study.

Second, findings may help guide the design of mental health counselling services that are feasible to implement, acceptable to adolescents, and relevant for their needs. As such, this study may help guide the implementation of South Africa’s mental health policy framework that focuses on expanding mental health service coverage to under-served populations, including at-risk adolescents. It will also assist the Western Cape Department of Health (WCDOH) in reaching its health service goals for 2030 by guiding decision-making about how to enhance service coverage to all populations in need of health care. Findings from the proposed study therefore have the potential to contribute to the transformation of the South African health care system. Further, if the ASPIRE intervention is shown to be feasible and has promising outcomes, it is likely to be applicable to other low-resourced settings.

Third, the proposed feasibility trial using a mixed-methods design will allow us to determine whether we can move forward to a larger effectiveness trial of the ASPIRE intervention. If our pre-specified qualitative or quantitative indicators highlight concerns regarding feasibility and acceptability impacting recruitment, retention, intervention delivery, data collection, randomization, blinding or safety, those relevant procedures will be modified accordingly. Should we find that significant modifications are required, we will consider the need for an internal pilot in the context of the full trial [66].

Despite these contributions and strengths of the proposed study, we anticipate some challenges to certain aspects of trial implementation. Recruitment of individual participants may pose challenges given that 15–17-year-olds will require parental consent. Previous research with alcohol use interventions reported lower rates of study enrolment and a bias towards participants with lower levels of alcohol use [67]. This was attributed to adolescents’ perception that their parents might learn about their alcohol use [63]. To address this challenge, parental consent is being sought after initial screening. Given that the inclusion criteria for study participation includes both depression and alcohol use, limited information on why the adolescent qualifies for the study will be disclosed. Further, retention of participants in a four-session intervention and through the lifespan of the study may pose some challenges. We have a comprehensive strategy to limit attrition (including reimbursement of transport costs) that has been used in other studies where we have obtained more than 80% follow-up rates [35].

In summary, this study has the potential to fill an important knowledge gap regarding promising trans-diagnostic counselling interventions for at-risk adolescents. Evidence generated by this study will be of direct relevance to current efforts to reform the public health system in South Africa and other LMICs where there is a focus on expanding access to mental health care to adolescents.

### Trial status

Recruitment for the trial began on 4 November 2019. Due to COVID-19, all non-essential community-based research was put on hold from 17 March 2020. Between 4 November 2019 and 18 March 2020, a total of 67 participants (33 treatment and 34 comparison) were recruited into the ASPIRE trial. Of these, 15 had completed all study activities by the time the study was paused. Seventeen participants in the intervention arm still had counselling sessions to complete, and 38 and 48 participants still required either 6-week or 3-month follow-up assessments, respectively. To reduce the risk of loss to follow-up, counselling sessions and follow-up assessments are currently being conducted telephonically for participants already recruited in the study. We are in the process of establishing a protocol to reduce the risk of COVID-19 transmission for when the ASPIRE trial is permitted to resume recruitment.

## Data Availability

Not applicable.
